# Modular co-culture engineering of *Yarrowia lipolytica* for amorphadiene biosynthesis

**DOI:** 10.1186/s12934-022-02010-0

**Published:** 2022-12-31

**Authors:** Monireh Marsafari, Fidelis Azi, Shaohua Dou, Peng Xu

**Affiliations:** 1 Department of Chemical, Biochemical and Environmental Engineering, University of Maryland Baltimore County, Baltimore, MD 21250 USA; 2 Department of Chemical Engineering, Guangdong Provincial Key Laboratory of Materials and Technologies for Energy Conversion (MATEC), Guangdong Technion – Israel Institute of Technology, Shantou, 515063 Guangdong China; 3 College of Life and Health, Dalian University, Dalian, 116622 Liaoning China; 4 Liaoning Marine Microorganism Engineering and Technology Research Center, Dalian, 116622 Liaoning China

**Keywords:** *Y. lipolytica*, Co-culture, Amorphadiene, Endoplasmic reticulum, Cellular localization

## Abstract

Amorphadiene is the precursor to synthesize the antimalarial drug artemisinin. The production of amorphadiene and artemisinin from metabolically engineered microbes may provide an alternate to plant secondary metabolite extraction. Microbial consortia can offer division of labor, and microbial co-culture system can be leveraged to achieve cost-efficient production of natural products. Using a co-culture system of *Y. lipolytica* Po1f and Po1g strains, subcellular localization of ADS gene (encoding amorphadiene synthase) into the endoplasmic reticulum, co-utilization of mixed carbon source, and enlargement of the endoplasmic reticulum (ER) surface area, we were able to significantly improve amorphadiene production in this work. Using Po1g/PPtM and Po1f/AaADSER_x3_/iGFMPDU strains and co-utilization of 5 µM sodium acetate with 20 g/L glucose in YPD media, amorphadiene titer were increased to 65.094 mg/L. The enlargement of the ER surface area caused by the deletion of the PAH1 gene provided more subcellular ER space for the action of the ADS-tagged gene. It further increased the amorphadiene production to 71.74 mg/L. The results demonstrated that the importance of the spatial localization of critical enzymes, and manipulating metabolic flux in the co-culture of *Y. lipolytica* can be efficient over a single culture for the bioproduction of isoprenoid-related secondary metabolites in a modular manner.

**Graphical Abstract:**

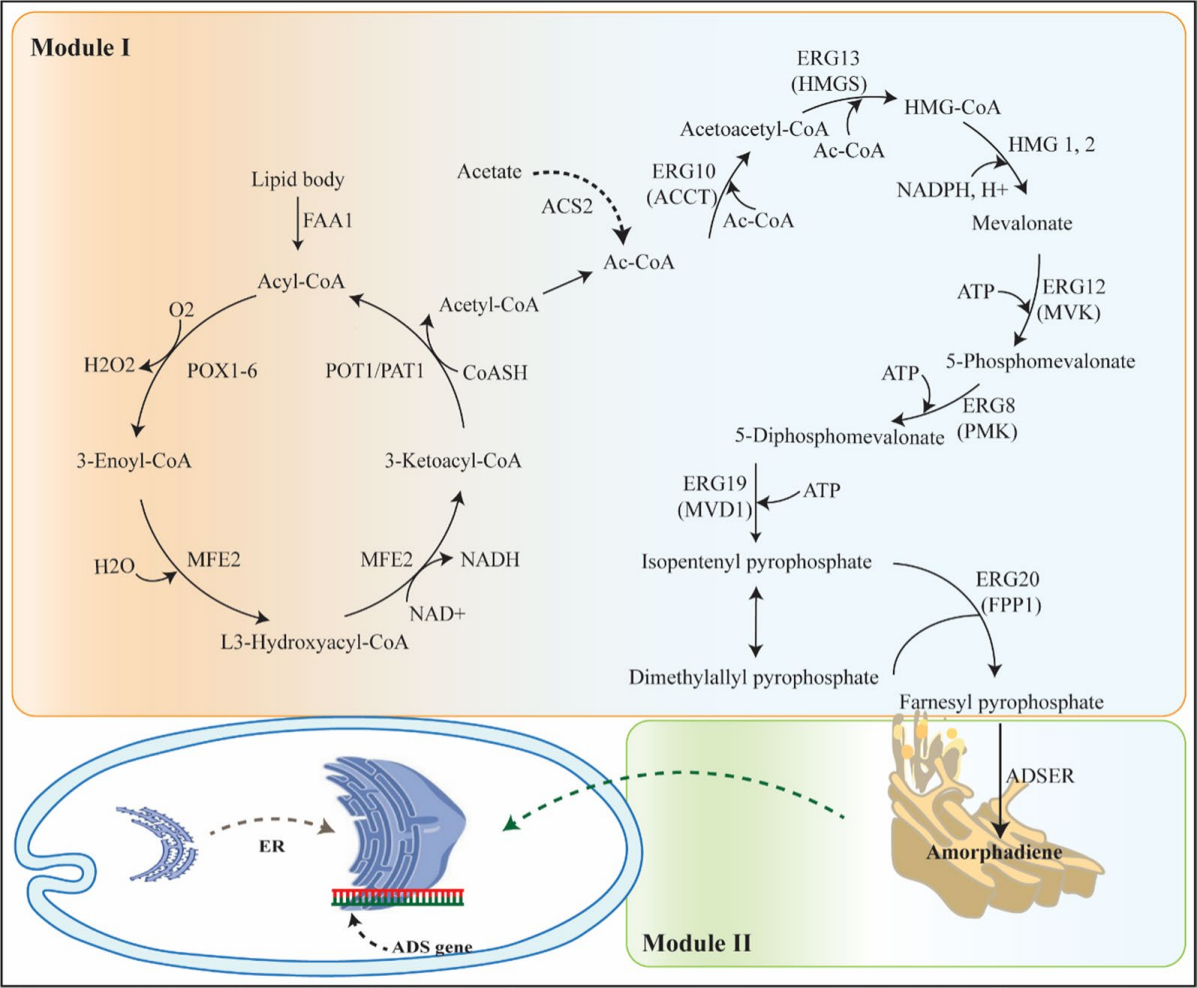

**Supplementary Information:**

The online version contains supplementary material available at 10.1186/s12934-022-02010-0.

## Background

Malaria affects half of the world’s population and constitutes one of the leading causes of death in developing countries. A parasite, *Plasmodium falciparum*, is the primary cause of malaria. Artemisinin is a sesquiterpene lactone isolated from the aerial parts of *Artemisia annua* L. [1, 2]. Artemisinin-based combination therapies (ACTs) are the most efficient treatment against malaria [3]. Beside the anti-malaria activity, artemisinin has been proven as an anticancer drug against colon, breast and prostate cancer [4–6], leukemia [7], and hepatitis B [8]. So far, the only commercial route for artemisinin supply is plant extraction, but unfortunately, the yield is low and not cost-effective [9]. Metabolic engineering and synthetic biology approach provide an alternative and scalable route for the heterologous bioproduction of interesting natural products in microbial hosts [10].

Amorphadiene is an olefin sesquiterpene; the first dedicated step to artemisinin synthesis in plants is by cyclization of farnesyl-pyrophosphate (FPP) mediated by amorphadiene synthase enzyme (ADS) [11]. Isopentenyl pyrophosphate (IPP) and its isomer dimethylallyl pyrophosphate (DMAPP) [12], which are biosynthesized from acetyl-CoA through mevalonate pathway, are converted to FPP by the action of the farnesyl pyrophosphate synthase (FPS) [9]. Geranylgeranyl diphosphate (GGPP) [12] is another intermediate in the mevalonate pathway that, in addition to participating in carotenoid biosynthesis, can also synthesize farnesyl diphosphate from geranyl diphosphate (GPP) and IPP [13]. Intending to provide direct and scalable access with cost that is comparable to the agriculture-sourced artemisinin, many researchers sought heterologous production of amorphadiene in various microbial hosts such as *Saccharomyces cerevisiae* [14, 15], *Escherichia coli* [9, 16, 17], *Bacillus subtilis* [18], *Azospirillum brasilense* [19], and *Yarrowia lipolytica* [20]. However, their attempts could not meet the increasing demand for artemisinin-based combination therapy (ACT) and amorphadiene/artemisinin is not yet commercially available [21].

*Yarrowia lipolytica* has been considered as a generally regarded as safe (GRAS) platform for the bioproduction of various natural products [22] with its high secretion capacity, strong acetyl-CoA and malonyl-CoA flux [23–26], and a large collection of genetic tools [25–28]. *Y. lipolytica* is a dimorphic, non-pathogenic ascomycetous yeast with a superior host for metabolic and genomic characteristics. It can be a great platform for cost-effective production of biochemicals derived from fatty acids, lipids, and acetyl-CoAs [29, 30]. In our previous study, by harnessing the innate mevalonate pathway, we introduced *Y. lipolytica* as a promising microbial host with a significant production capacity of amorphadiene in a monoculture [20].

In addition to the many metabolic engineering strategies to improve the bioproduction of a wide variety of value-added biochemicals through a single microbial host, this methodology is still challenging. Increased requirements for fulfilling complicated biosynthetic pathways and reaching efficacy are two critical issues facing monoculture fermentations. They have been considerably circumvented by employing modular co-culture approaches [31, 32]. With manipulating the mixture of compatible hosts and nutrients, co-culture strategy provides a platform that absorbs cheaper substrates, improves cell growth without complex treatments, and extends the yield and spectrum of final product/s [33]. The optimized *Y. lipolytica- Chlorella pyrenoidosa* co-culture increased carbon and nitrogen assimilation and drove the carbon flow to a higher yield of microbial biomass such as lipid, carbohydrates, and protein [34]. Consuming lactate as a carbon source and decreasing lactic acid in the media, *Y. lipolytica* participated as a partner accelerated the *Lactococcus lactis* growth and accumulation of nisin up to 50% in *Y. lipolytica–L. lactis* co-culture [35].

To expand the biosynthesis of amorphadiene, a co-culture of two *Y. lipolytica* strains, Po1g, and Po1f, was investigated in this study. Interaction between two strains in this study is considered commensalism as Po1g strains provided the substrate for Po1f strains and helped to improve the amorphadiene titer [36]. The mevalonate pathway was divided into two strains to facilitate the conversion of glucose to amorphadiene. Although *Y. lipolytica* produces a high amount of acetyl-CoA and malonyl-CoA, our previous attempt demonstrated that harnessing lipogenic acetyl-CoA pathway could improve the bioproduction of amorphadiene [20]. So, we first engineered the Po1g strain to improve its secretion capacity. Then to maximize the HMG-CoA pathway intermediate uptake and reduce the byproduct production, the amorphadiene synthase (ADS) was localized in the endoplasmic reticulum (ER) [37]. In addition to studying multiple sugar sources, the enlargement of the endoplasmic reticulum was investigated to provide a larger cellular space area to improve the amorphadiene bioproduction. This research will provide more comprehensive insight into the application of *Y. lipolytica* co-culture to promote cost-effective bioproduction of natural products.

## Material and method

### Genes, plasmids, and strains

Genes encoding *A. annua* L. amorphadiene synthase (AaADS), *Y. lipolytica* 3-ketoacyl-CoA thiolase (YlPOT1), *Y. lipolytica* Acetyl-CoA C-acetyltransferase (YlPAT1), *Y. lipolytica* farnesyl pyrophosphate [14], *Y. lipolytica* mevalonate diphosphate decarboxylase (YlMVD1), truncated form of *Y. lipolytica* 3-hydroxy-3-methylglutaryl-CoA reductase (tYlHMG1), *Y. lipolytica* phosphomevalonate kinase (ERG8), and *Y. lipolytica* mevalonate kinase (ERG12) were reported in our previous report [20]. *Y. lipolytica* geranylgeranyl diphosphate synthase (YlGGPP), *Y. lipolytica* sterol-regulatory element-binding protein (YlUPC-2), *Y. lipolytica* multifunctional beta-oxidation enzyme (YlMFE2), and *Y. lipolytica* acetyl-CoA synthetase (YlACS2) were amplified from *Y. lipolytica* Po1g genomic DNA by PCR reaction. *By PCR reaction, Escherichia coli farnesyl diphosphate synthase (EcispA) was amplified from E. coli BL21 genomic DNA*. All the genes included in this paper are listed in Additional file 1: Table S1.

Plasmid pYLXP’ [38], prDNA1, and prDNA2 [25] were previously designed and maintained in our laboratory. *E. coli* strain NEB5α was used for plasmid construction and maintenance. This strain was grown in LB liquid media or plate containing 15 g/L agar at 37 °C supplemented with 100 µg/mL ampicillin for selection. *Y. lipolytica* strains Po1g∆Lue, and Po1f∆Lue∆Ura were used as chassis to produce amorphadiene. All strains used in this research were listed in Additional file 1: Table S2.

### Pathway construction and molecular cloning

YlGGPP, YlUPC-2, YlMFE2, YlACS2, and EcispA genes were obtained by PCR reaction and using specifically optimized primer by integrated DNA Technologies Company, USA (Additional file 1: Table S3). Before designing primer and amplifying genes, any detected internal introns in the genes were removed. USING THE GIBSON ASSEMBLY METHOD (New England Biolabs), the PCR product was introduced at the SnaBI and KpnI digestion site of pYLXP’, prDNA1, and prDNA2. To complete the intron fragment, the sequence of “TAACCGCAG” was replaced with the start codon of each gene [27].

The YaliBrick standard was used to assemble the amorphadiene pathway into the designed plasmids [27]. T4 ligation was used to assemble recipient and donor plasmids and prepare monocistronic cassettes. For screening and validation of the desired cassettes containing multiple gene pathways, the *E. coli* strain NEB5α transformation and mostly KpnI/XhoI digestion were used. All the plasmids used in this research are listed in Additional file 1: Table S4.

*Yarrowia lipolytica* transformation was completed by using the lithium acetate (LiAc) method as described previously [20].

### Acetyl-CoA assay

Desired recombinant *Y. lipolytica* strains were grown and used to prepare the pre-culture. The pre-culture was diluted into fresh 50 mL media to prepare the secondary culture with the OD600 of 0.05. The secondary culture was grown until the OD600 reached 0.4; the cells were harvested by centrifuging at 12,000 rpm for 5 min. Subsequently, 10 mL of pre-chilled (− 80 °C) methanol was added to each sample to quench cell metabolism and then centrifuged at 12,000 rpm for 5 min to remove the supernatant. Immediately 2 mL of boiling ethanol was added to cell pellets and then boiled for an additional 15 min. The mixture was thoroughly treated by glass beads for 5 min (vortex) to release intracellular metabolites. After centrifugation, the supernatant was vacuum dried and resuspended into 200 µL ddH_2_O. The resultant solution was analyzed using the acetyl-Coenzyme A Assay Kit (Sigma-Aldrich, USA). The concentration of acetyl-CoA was obtained from a standard curve. The reported acetyl-CoA represents the 50× concentrated sample (10 mL culture with OD 0.4 concentrated to 200 µL sample).

### Enlargement of the ER by deletion of PAH1 gene

All primers used in this research were listed in Additional file 1: Table S3. The PAH1 gene was deleted through the homologous recombination method. To construct a cassette for the deletion of PAH1, a primer set of pah1upfw and pah1uprv was used to PCR amplify of 1000 bp fragment immediately upstream from the start codon PAH1 using genomic Po1f DNA as template. Another primer set of pah1dwfw and pah1dwrv was used to amplify 1000 bp fragment immediately downstream from the stop codon. These two upstream and downstream fragments were size verified via gel electrophoresis and purified using ZYMO Clean and Concentrator kits. The purified upstream fragment was Gibson assembled with the linearized and gel purified pYLXP’-ylURA3 backbone at the ClaI digestion site. The pYLXP’-ylURA3 plasmid was previously constructed and maintained in our laboratory. Colonies were verified via colony PCR using pah1upfw and tef_rv primers.

Positive colonies were inoculated into LB media containing ampicillin for overnight culture. The plasmid was purified using ZYMO Miniprep kits, and Sanger sequenced by QuintaraBio. The downstream 1000 bp fragment was cloned into the SalI site of pYLXP’-ylURA3-PAH1UP to yield pYLXP’-ylURA3-PAH1. The primers xpr2_fw and pah1dwrv were used for colony PCR and sanger sequencing of positive colonies containing 1000 bp of the downstream fragment. The sequencing-verified pYLXP’-ylURA3-PAH1 was used as template to amplify a deletion cassette of PAH1 using primers pah1casfw and pah1casrv. The size verified PCR product was subsequently digested with DpnI to remove the template plasmid.

The PAH1 knockout cassette was transformed into mutant strain Po1f∆DGA_1,2_, which was previously prepared and maintained in our laboratory, using the hydroxyurea-based protocol to enhance homologous recombination [39, 40]. Transformants were plated onto CSM-Ura plates. These preliminarily positive transformants were suspended into 4 µL 0.02 M sodium hydroxide and boiled at 95 °C for 10 min to lyse the transformant yeast cells. Two colony PCR reactions were performed labeled A and B for each colony. The primers pah1upchkf and tef_rv were used for the colony PCR of group A, while the primers pah1dwchkr and xpr2_fw were used for the group B. The size verified colonies were selected for subsequent transformation and amorphadiene production.

### Growth condition and fermentation cultivation

For the pre-culture, single colonies of each transformant were inoculated from fresh plates in 3 mL YPD media and grown at 30 °C and 250 rpm agitation for 48 h. The monocultures were carried out in 250 mL flasks with 40 mL working volume and cultivated at 30 °C and 250 rpm agitation. For co-culture, the equal volumes of pre-culture materials (1.6% v/v) were mixed and inoculated into 250 mL flasks with 40 mL working volume and cultivated at 30 °C and 250 rpm agitation for 144 h. YPD containing glucose was used as a basic media for the fermentation, and it was manipulated in three other groups to carry out the co-culture procedure. In the first group, YPD containing 20 g/L glucose was supplemented with 0, 5, 10, and 15 µM sodium acetate as a secondary carbon source and enriched the media. In the second group, YPD containing 20 g/L glucose was supplemented with 0, 25, 50, and 75 µM acetic acid. The equal mixture (1:1 v/v) of glucose and xylose, 20 g/L, was investigated for the third group. The cultures were overlaid with 20% (v/v) dodecane after 48 h of incubation to trap amorphadiene.

### Analytical method

All the experiments were carried out in triplicate. All samples were analyzed based on our previously reported method [20].

## Results and discussion

### Boosting acetyl-CoA production

Acetyl-CoA is an important precursor for various bioproducts, including isoprenoids, flavonoids, and sesquiterpenes. In yeasts, acetyl-CoA is biosynthesized in the different compartments such as mitochondria, peroxisome, and cytosol [41]. The mitochondrial acetyl-CoA needs to be transported out of mitochondria by acetyl-carnitine shuttle to be used by cytosolic reaction [42]. While in *Y. lipolytica*, most of the cytosolic acetyl-CoA is directed toward lipid biosynthesis. The cellular fatty acid is degraded into acetyl-CoA units through β-oxidation and by the action of fatty acyl-CoA synthetase [41], multifunctional enzyme type 2 (MFE2), 3-ketoacyl-CoA thiolase (POT1), and Acetyl-CoA C-acetyltransferase (PAT1) in that sequence.

**Fig. 1:**
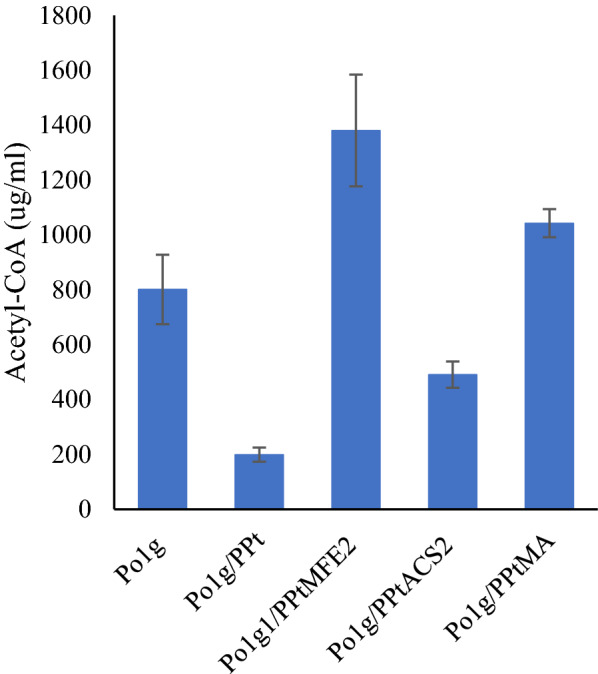
Boosting intracellular acetyl-CoA by using different strategies. PPt: construct containing PAT1, POT1, and tHMG1 genes; PPtMFE2: construct containing PAT1, POT1, tHMG1, and MFE2 genes; PPtACS2: construct containing PAT1, POT1, tHMG1, and ACS2 genes; PPtMA: construct containing PAT1, POT1, tHMG1, MFE2 and ACS2 genes. Detailed gene annotation can be found in Additional file 1: Table S2

Our previous study demonstrated that by harnessing the lipogenic acetyl-CoA pathway and overexpression of PAT1 and POT1, bioproduction of amorphadiene can be improved. Here we first introduced the construct containing tYlHMG1, PAT1, and POT1 into *Y. lipolytica* Po1g strain and measured the acetyl-CoA concentration in the culture. Interestingly, the acetyl-CoA production was reduced compared to the control Po1g. Next, we added MFE2 and ACS2 genes separately, combined with this strain, and tested the acetyl-CoA level. These three strains produced a higher acetyl-CoA than Po1g/PPt (Fig. 1). Interestingly, Po1g/PPtMFE2 significantly produced a higher titer of acetyl-CoA up to 1380 ± 398 μg/mL compared to all the other strains (Fig. 1). To use the strain for co-culture consortia, we introduced the PPtMFE2 construct into the prDNA1 plasmid to be integrated into the genome of the Po1g strain. It should be noted that our acetyl-CoA level was determined as 34.09 µM in the cell culture when considering the concentration factor (50×). When this number is normalized by the amount of cell (10 mL cell with 0.4 OD), it roughly gives about 0.213 µmole/gDCW of acetyl-CoA (1 OD is about 0.4 gDCW/L biomass), which is comparable to the reported data.

### Sequestrating amorphadiene synthase to ER

Our first step to establishing robust microbial cells for amorphadiene production requires effective expression of plant-derived ADS. Plant enzymes are often poorly expressed in heterologous hosts [18]. For this, improving the expression of ADS plays a pivotal role in increasing amorphadiene bioproduction. This can be done by modifying the N-terminal of proteins through fusion protein partner to improve the ADS translation and expression.

Furthermore, HMG1 (HMG-CoA reductase) is the rate-limiting enzyme of the sterol biosynthesis pathway and converts HMG-CoA to mevalonate [37]. Squalene synthase (SQS1) converts two identical molecules of farnesyl pyrophosphate (FPP) to squalene. Both HMG1 and SQS1 are localized in ER, indicating the sterol biosynthetic pathway is spatially organized, and ER may provide the optimal microenvironment for their catalytic efficiency. Biochemical studies and metabolite profiling indicate that squalene synthase is the major competing step for amorphadiene production and its downregulation leads to improved amorphadiene bioproduction [43]. It was found that the C-terminal residues of SQS1 are responsible for targeting the ER of yeast [44]. To maximize the activity of amorphadiene synthase, we fused the SQS1-ER localization domain with the C-terminal of ADS using a glycine linker. For this, we amplified the SQS1 domain using TransEr_F and TransEr_R primer pairs and linked the PCR product to ADS gene using Gibson assembly, and cloned the hybrid ADS into digested prDNA2 plasmid at the SnaBI and KpnI restriction site. This chimeric protein maintains ADS activity and effectively competes with squalene synthase for farnesyl pyrophosphate (FPP), leading to the synthesis of about 15.47 mg/L amorphadiene (Additional file 1: Fig. S1). The localization of ADS to ER may serve as a metabolic channel for HMG1-CoA and FPP, which effectively converts HMG-CoA to amorphadiene. Furthermore, the spatially organized protein may prevent intermediates diffusion, concentrate the critical metabolites and increase the catalytic activity.

In our previous study, increased AaADS copy number improved the amorphadiene titer [20]. Hence, we increased the AaADSER gene into plasmid up to three copy numbers for co-culture. Furthermore, we integrated prDNA2-AaADSER_x3_ plasmid into the Po1f genome for all sections of this study and subsequently transformed it with pYLXP’-EcispA-YlGGPP-YlERG20-YlERG8-YlERG12-YlMVD1-YlUPC2 plasmid if required with the aim of mevalonate optimization.

### The effect of glucose co-utilization with acetate on amorphadiene titration

Acetate plays a multi-dimensional role in living cells. It can induce sporulation of fungi, yeast, and bacteria as a sole carbon source in growth media. On the other hand, it can inhibit the microorganism’s growth when used as a preserving additive in the food industry. The sensitivity of *Y. lipolytica* strain to acetate depends on the growth conditions. *Y. lipolytica*, the co-utilization of acetate and glucose stimulate the wild strain growth [45]. The use of acetate as a secondary carbon source for *Y. lipolytica* is promising due to its flux capacity for acetyl-CoA that is confirmed as a rate-limiting step for amorphadiene bioproduction.

**Fig. 2:**
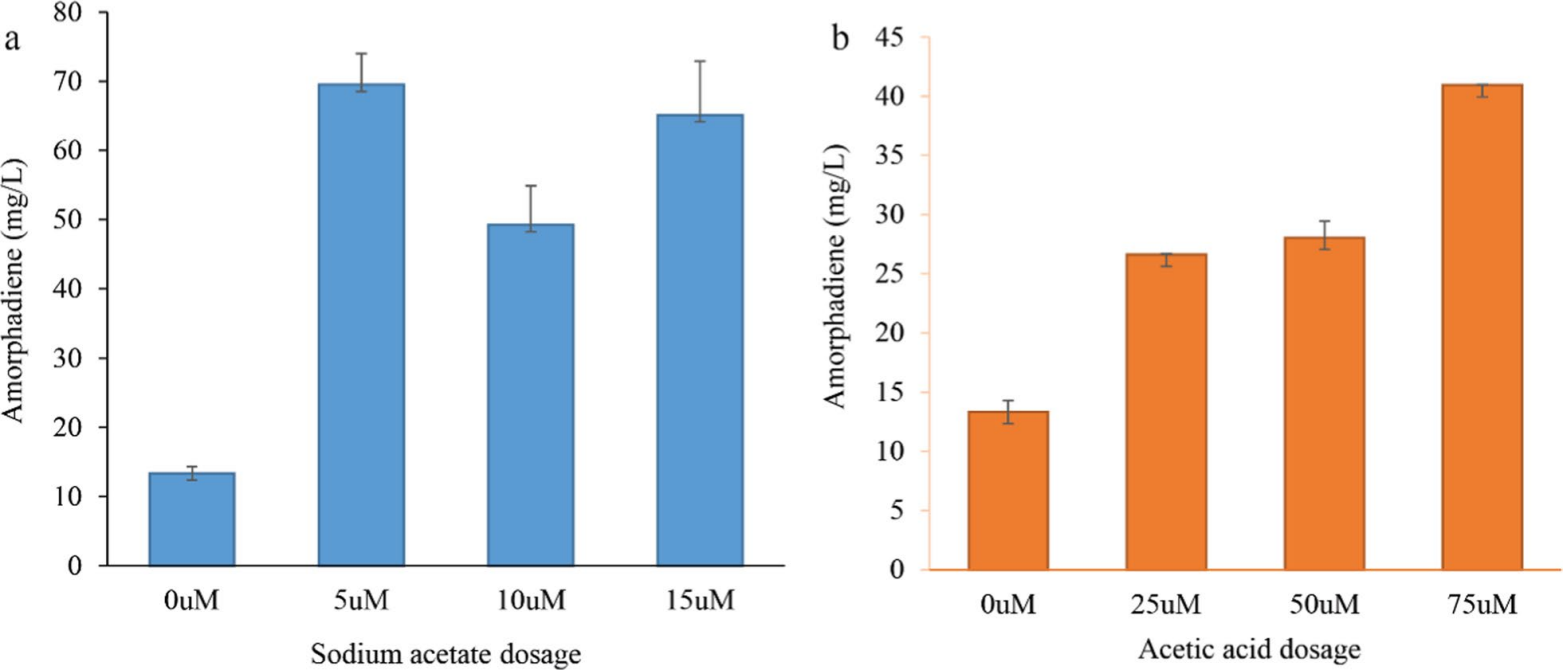
The effect of glucose co-utilization with sodium acetate and acetic acid on amorphadiene production in a co-culture system

Hence, we studied the co-utilization of acetic acid and sodium acetate in the YPD media containing glucose. For this, we investigated 0, 5, 10, and 15 µM of sodium acetate and 0, 25, 50, and 75 µM of acetic acid to find the best dosage for the co-culture of Po1g/PPtM and Po1f/ADER_x3_/iGFMPDU strains. As illustrated in Fig. 2, 5 µM of sodium acetate produced the maximum amount of amorphadiene up to 69.503 mg/L in our tested co-culture strain. In contrast, the co-utilization of acetic acid and glucose with the highest titration of 40.951 mg/L had no significant effect on amorphadiene production (Fig. 2b). The reason that sodium acetate (NaAc) can serve as better co-substrate lies in the fact that it can buffer the media pH due to the basic nature of NaAc, compared to the acidic nature of acetic acid with a pKa around 4.76.

### The effect of xylose co-utilization with glucose on amorphadiene production

**Fig. 3:**
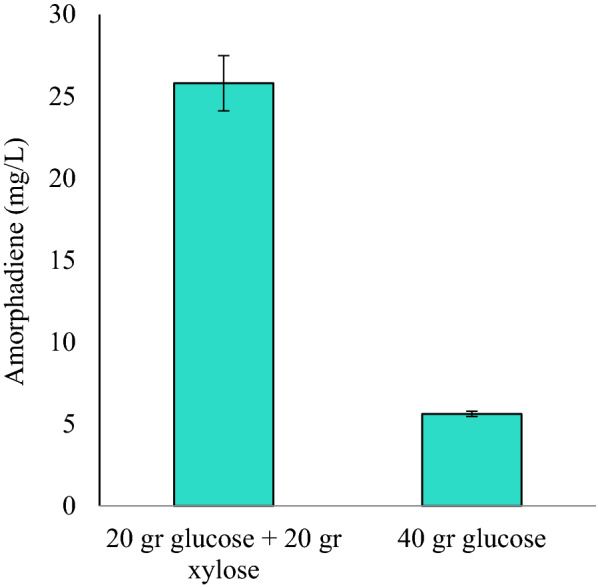
Glucose co-utilization with xylose affects amorphadiene production in a co-culture system

Xylose is the most prevalent sugar source obtained from hemicellulosic hydrolysis of sugar cane bagasse and is of great interest for a green economy. *Y. lipolytica* lacks an effective metabolic pathway for xylose uptake. Efforts have been made to engineer strains that can use xylose as a sole carbon source in the media [46]. Due to the ability of engineered strains in a co-culture system to uptake and deal with the mixed feedstock in media and the weakness of *Y. lipolytica* to uptake xylose as a sole sugar source, we studied co-utilization of 20 g/L xylose with 20 g/L glucose in our co-culture system. Glucose as a sole carbon source in the medium was used as the control. The results indicated that the mixture of xylose and glucose for the co-culture of Po1f/ADER_x3_/iGFMPDU and Po1g/PPtM improved the amorphadiene titer up to 25.802 mg/L and 4.59-fold compared to the control sample (Fig. 3). Interestingly, the maximum titer of amorphadiene in both mix and single sugar sources was lower than the amount obtained from co-utilization of glucose with sodium acetate. This may be due to the innate endogenous xylulokinase (XK) gene that limits the growth in *Y. lipolytica* in the presence of glucose. On the other hand, this result suggests that *Y. lipolytica* has superior capacity to assimilate and digest acetate, and acetate served as a metabolic shortcut to acetyl-CoA and HMG-CoA, which improves amorphadiene production.

### Boosting amorphadiene titer by disrupting PAH1 gene

*Yarrowia lipolytica* PAH1 gene is responsible for a phosphatidic acid de-phosphorylase enzyme (PAP). PAH1 dephosphorylates phosphatidic acid to diacylglycerols (DAGs). Then DAG is combined with acyl-CoA to form triacylglycerols. It was confirmed that deletion of *Y. lipolytica* PAH1 gene resulted in the accumulation phospholipids in the cells which ultimately led to an increase in the size of ER [47]. To produce a specialized *Y. lipolytica* strain that can be used in a co-culture system with a sole sugar source and provide the maximum ER membrane space for the action of the ADSER gene, we knocked out the PAH1 gene through the conventional homologous recombination using a *URA3* disrupting cassette.

**Fig. 4:**
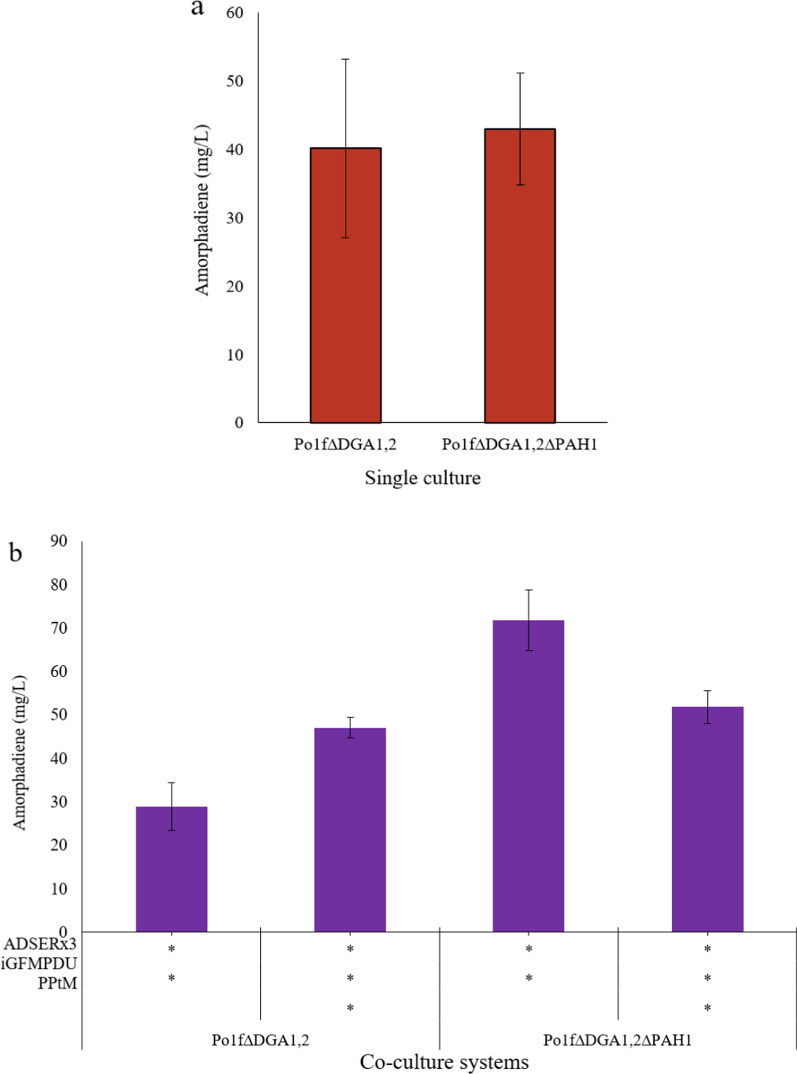
The effect of PAH1 disruption on amorphadiene titer in single strain fermentation and co-culture systems. **a** Amorphadiene titer in single culture fermentations. **b** Amorphadiene titration through co-culture of Po1g and Po1f strains. ADSERx3: construct containing three copy number of ADSER gene; iGFMPDU: construct containing ispA, GGPP, ERG20, ERG12, ERG8, MVD1, and UPC2 genes, PPtM: construct containing PAT1, POT1, tHMG1, and MFE2 genes

To modify the PAH1 gene, we used Po1f∆DGA_1,2_ strain deficient of both DGA1 and DGA2 steps that was previously constructed in our laboratory. Using Po1f∆DGA_1,2_ and Po1f∆DGA_1,2_∆PAH1 strains, we designed four commensalism co-culture systems (Fig. 4b) and two single culture fermentations (Fig. 4a) to study the effect of PAH1 deletion on amorphadiene titer. We observed that the two strains transformed with AaADSER_x3_ construct in single culture and produced more than 40 mg/L amorphadiene, which is a valuable amount obtained only with PAH1 deletion and in single culture (Fig. 4a). Next, we designed commensalism co-culture systems consisting of specialized Po1f∆DGA_1,2_ and Po1f∆DGA_1,2_∆PAH1 strains with genome integration of prDNA2-AaADSER_x3_ plasmid, and strains Po1g/iGFMPDU, and Po1g/PPtMA strains. Our results indicated that this co-culture system significantly improved amorphadiene titer with the maximum amount of 71.74 mg/L and using glucose as a sole sugar source in the medium (Fig. 4b). Modifying the PAH1 gene increased amorphadiene titer in a co-culture system, but interestingly, Po1g/PPtM did not significantly improve amorphadiene titer. This may be because the simultaneous modification of DGA_1,2_ and PAH1 accelerated acetyl-CoA utilization, which was competing for the amorphadiene biosynthesis pathway. At the same time, the Po1g/PPtM strain produces more acetyl-CoA but defects in *Y. lipolytica* growth.

## Conclusion

Recently, co-culture system has been established as one of the most powerful tools in improving the production of valuable biochemicals. One of the criteria for selecting a host in co-culture is its inability to consume, degrade, and inactivate the final product and increase the end-product yield. In this report, we demonstrated that *Y. lipolytica* is a fascinating heterologous host for amorphadiene production, due to their innate mevalonate pathway and high level of cellular acetyl-CoA. One of the first steps in designing a successful co-culture system is to engineer the microbial workhouse with the modular pathways to optimize the final product. In the co-culture system, the constructed specialist strains could deal with fluctuations in feedstock compositions, while the single recombinant strain was unable to do this. Utilizing *Y. lipolytica*’s ability to grow well on various carbon sources such as glucose, xylose, acetate, ethanol, and glycerol would have a tremendous impact on the bioproduction of value-added biochemicals, potentially reducing their cost of bioproduction. On the other hand, acetate can be directly converted to acetyl-CoA, and this metabolic shortcut can be an asset to accelerate the accumulation of natural products in single or co-culture fermentations. In this research, we assembled three constructs for β-oxidation and mevalonate pathway and localized amorphadiene synthase in ER. Next, we attempted to study the effect of co-utilizing acetate and xylose as secondary carbon sources with glucose in the medium to divide the metabolic functions between strains. It is likely that mevalonate is the intermediate compound that coordinates the metabolic reactions in the co-culture system. We also modified the PAH1 gene to enlarge the ER surface area. Interestingly, ∆PAH1 strain providing enough acetyl-CoA for amorphadiene biosynthesis eliminated the need for the construct responsible for optimizing the β-oxidation pathway. The ∆PAH1 strain provided enough membrane space for the maneuver of ADSER enzyme and the utilization of FPPs toward amorphadiene synthesis. The applied factors for co-culture optimization removed the metabolic bottlenecks, increased amorphadiene titer to 71.74 mg/L, using glucose as the sole carbon source. This study demonstrates that *Y. lipolytica* can be a superior host for modular co-culture and can be used to improve the bioproduction of amorphadiene and other value-added metabolites.

## Supplementary Information

**Additional file 1:Table S1.** Genes used in this paper. **Table S2.** Strains constructed in this paper. **Table S3.** Primers used in this paper. **Table S4.** Plasmids used in this paper. **Figure S1.** Amorphadiene production through original and ER-tagged ADS gene.

## Data Availability

The datasets during and/or analysed during this study are available from the corresponding author on reasonable request.
