# Supplementary material for: Modular co-culture engineering of Yarrowia lipolytica for amorphadiene biosynthesis
Source: Microb Cell Fact. 2022 Dec 31;21:279. doi: 10.1186/s12934-022-02010-0 (PMC9805133; doi:10.1186/s12934-022-02010-0)
Supplement: Supplementary file 1 — Additional file 1:Table S1. Genes used in this paper. Table S2. Strains constructed in this paper. Table S3. Primers used in this paper. Table S4. Plasmids used in this paper. Figure S1. Amorphadiene production through original and ER-tagged ADS gene. [file 12934_2022_2010_MOESM1_ESM.docx]

**Modular co-culture engineering of *Yarrowia lipolytica* for amorphadiene biosynthesis**

**Monireh Marsafari^1^, Fidelis Azi^2^, Shaohua Dou^3,4^*, Peng Xu^1, 2^***

^1^Department of Chemical, Biochemical and Environmental Engineering, University of Maryland Baltimore County, Baltimore, MD, 21250, USA

^2^Department of Chemical Engineering, Guangdong Provincial Key Laboratory of Materials and Technologies for Energy Conversion (MATEC), Guangdong Technion – Israel Institute of Technology, Shantou, Guangdong 515063, China

^3^College of Life and Health, Dalian University, Dalian, Liaoning 116622, China

^4^Liaoning Marine Microorganism Engineering and Technology Research Center,

Dalian, Liaoning 116622, China

*Corresponding author: Email: [peng.xu@](mailto:peng.xu@gtiit.edu.cn)gtiit.edu.cn (PX) and doushaohua@dlu.edu.cn (SD). Phone 86-754-88077163

**Table S1. Genes used in this paper.**

| Gene | Origin | Database and accession number | Optimization | References |
| --- | --- | --- | --- | --- |
| *AaADS* | *Artemisia annua* | NCBI: AEQ63683.1 | Yes | (Marsafari & Xu, 2020) |
| *HMG1* | *Yarrowia lipolitica* Po1g | UniProtKB: Q6C704 | Yes | (Huang et al., 2018) |
| *GGPP* | *Yarrowia lipolitica* Po1g | UniProtKB: Q9c452 | Yes |  |
| *UPC2* | *Yarrowia lipolitica* Po1g | UniProtKB: Q12151 | Yes |  |
| *MFE2* | *Yarrowia lipolitica* Po1g | UniProtKB: Q9P4D9 | Yes |  |
| *ACS2* | *Yarrowia lipolitica* Po1g | UniProtKB: Q6C2Q5 | Yes |  |
| *ispA* | *Escherichia coli* | NCBI: 945064 | Yes |  |
| *ERG8* | *Yarrowia lipolitica* Po1g | GRYC: YALI0E06193g | Yes | (Martin et al., 2003) |
| *ERG12* | *Yarrowia lipolitica* Po1g | GRYC: YALI0B16038g | Yes | (Martin et al., 2003) |
| *MVD1* | *Yarrowia lipolitica* Po1g | GRYC: YALI0F05632g | Yes | (Martin et al., 2003) |
| *ERG20* | *Yarrowia lipolitica* Po1g | GRYC: YALI0E05753g | Yes | (Westfall et al., 2012) |
| *POT1* | *Yarrowia lipolitica* Po1g | GRYC: YALI0E18568g | Yes |  |
| *PAT1* | *Yarrowia lipolitica* Po1g | GRYC: YALI0E11099g | Yes |  |

(1) GRYC refers to the genome resources for yeast chromosomes (http://gryc.inra.fr/index.php?page=home).

**Table S2. Strains constructed in this paper.**

| Strain | Annotation | References |
| --- | --- | --- |
| Po1f/ADSER_x3_^1^ | Po1f containing genome integrated prDNA2-AaADSER_x3_, used for the optimization of amorphadiene production | This work |
| Po1f/ADSER | Po1f containing prDNA2-AaADSER, used for the optimization of amorphadiene production | This work |
| Po1f/ADS | Po1f containing prDNA2-AaADS, used for the optimization of amorphadiene production | This work |
| Po1f∆DGA_1,2_/ADSER_x3_ | Po1f∆DGA_1,2_ containing prDNA2-AaADSER_x3_, used for the optimization of amorphadiene production | This work |
| Po1f∆DGA_1,2_∆PAH1/ADSER_x3_ | Po1f∆DGA_1,2_ containing prDNA2-AaADSER_x3_, used for the optimization of amorphadiene production | This work |
| Po1g/PPt | Po1g containing pYLXP’-YlPAT1-YlPOT1-YltHMG1, used for the optimization of acetyl-CoA production | This work |
| Po1g1/PPtMFE2 | Po1g containing pYLXP’-YlPAT1-YlPOT1-YltHMG1-YlMFE2, used for the optimization of acetyl-CoA production | This work |
| Po1g/PPtM | Po1g containing genome integrated prDNA1-YlPAT1-YlPOT1-YltHMG1-YlMFE2, used for the optimization of acetyl-CoA production | This work |
| Po1g/PPtACS2 | Po1g containing pYLXP’-YlPAT1-YlPOT1-YltHMG1-YlACS2, used for the optimization of acetyl-CoA production | This work |
| Po1g/PPtMA | Po1g containing pYLXP’-YlPAT1-YlPOT1-YltHMG1-YlMFE2-YlACS2, used for the optimization of acetyl-CoA production | This work |
| Po1g/iGFMPDU | Po1g containing pYLXP’- EcispA-YlGGPP-YlERG20-YlERG12-YlERG8-YlMVD1-YlUPC2, used for the optimization of mevalonate pathway | This work |
| Po1f/ADSER_x3_/iGFMPDU | Po1f containing both prDNA2- AaADSERx3 and pYLXP’- EcispA-YlGGPP-YlERG20-YlERG12-YlERG8-YlMVD1-YlUPC2 used for the optimization of amorphadiene production | This work |
| Po1f∆DGA_1,2_/ADSER_x3_/ iGFMPDU | Po1f∆DGA_1,2_ containing prDNA2-AaADSER_x3_ and pYLXP’- EcispA-YlGGPP-YlERG20-YlERG12-YlERG8-YlMVD1-YlUPC2 used for the optimization of amorphadiene production | This work |
| Po1f∆DGA_1,2_∆PAH1/ADSER_x3_/iGFMPDU | Po1f∆DGA_1,2_∆PAH1 containing prDNA2-AaADSER_x3_ and pYLXP’- EcispA-YlGGPP-YlERG20-YlERG12-YlERG8-YlMVD1-YlUPC2 used for the optimization of amorphadiene production | This work |

The subscripts “_x3_” refer to gene copy number in the plasmids.

**Table S3. Primers used in this paper.**

| Primer | Sequence (5’-3’) |
| --- | --- |
| Trans_Er F | GGAGGTGGAGGTTCAACCTTCACTTTCTGCGCC |
| Trans_Er R | GGACAGGCCATGGAACTAGTCGGTACCCTAATCTCTCAGAGGAAACATC |
| AaADS F | CCGACCAGCACTTTTTGCAGTACTAACCGCAGTCATTGACAGAGGAAAAGCCTA |
| AaADS R | GAAAGTGAAGGTTGAACCTCCACCTCCTATGGACATGGGATAAACAAGAAG |
| GGPP F | AGCACTTTTTGCAGTACTAACCGCAGGATTATAACAGCGCGGATTTCAAG |
| GGPP R | GTGGGGACAGGCCATGGAACTAGTCGTCACTGCGCATCCTCAAAGTACTT |
| UPC2 F | CCGACCAGCACTTTTTGCAGTACTAACCGCAGGCTCTGCAAGACCGACAACCCTAC |
| UPC2 R | GGACAGGCCATGGAACTAGTCGGTACCCTATTCTCGAGGAGAGTGGATCTT |
| MFE2 F | CCAGCACTTTTTGCAGTACTAACCGCAGTCTGGAGAACTAAGATACGAC |
| MFE2 R | GGACAGGCCATGGAACTAGTCGGTACCTTAGAGCTTAGCATCCTTGGGGA |
| ACS2 F | CCGACCAGCACTTTTTGCAGTACTAACCGCAGTCTGAAGACCACCCAGCCA |
| ACS2 R | GGACAGGCCATGGAACTAGTCGGTACCTTACTTTTTCAACGAGTGAACAA |
| EcispA F | GACCAGCACTTTTTGCAGTACTAACCGCAGGACTTTCCGCAGCAACTCGA |
| EcispA R | GCCATGGAACTAGTCGGTACCTTATTTATTACGCTGGATGATGTAG |
| pah1upfw | GGCATCCCTAAATTTGATGAAAGCCTAGGAAGCTGTTGTGGCCTCACTCTCA |
| pah1uprv | ATAATGTATGCTATACGAAGTTATGGGCAGCTCGGTCTTGGTGC |
| pah1dwfw | GCTAGCGAGACAATAACGGAGGAATAGGAAATCAAGCATAAATATATCCGTACTGTAC |
| pah1dwrv | ATGTTACATCCTTTTATCAGACATAGTCGACAGTTCACTTGAACCTGCTTGGCCAAA |
| pah1upchkf | CCCAGCCGGCTATACTTGAACATC |
| pah1dwchkr | AGTGGAAACTGGTTTTGGACACCTGT |
| pah1casfw | AAGCTGTTGTGGCCTCACTCTCA |
| pah1casrv | AGTTCACTTGAACCTGCTTGGCCAAA |
| tef_rv | GTAGTCTATTTTGCGTCCGGCATGG |
| xpr2_fw | TTGCCTACAACGGTGTTGGCAT |
| Tef_F | GCAGTCTGGAATCTACGCTTGTTCA |
| Xpr2_rv | GCCTAAATGCCAACACCGTTGTAGGC |

**Table S4. Plasmids used in this paper.**

| Plasmid | Annotation |
| --- | --- |
| pYLXP' | YaliBrick plasmid, used for pathway assemble |
| prDNA1 | YaliBrick plasmid, used for pathway assemble |
| prDNA2 | YaliBrick plasmid, used for pathway assemble |
| prDNA2-AaADS | For the construction and optimization of amorphadiene production |
| prDNA2-AaADSER | For the construction and optimization of amorphadiene production |
| prDNA2-AaADSER_x3_^1^ | For the construction and optimization of amorphadiene production |
| pYLXP'-tYlHMG1-YlPOT1-YlPAT1 | For the construction and optimization of acetyl-CoA biosynthesis |
| prDNA1-tYlHMG1-YlPOT1-YlPAT1 | For the construction and optimization of acetyl-CoA biosynthesis |
| pYLXP'- tYlHMG1-YlPOT1-YlPAT1-YlMFE2 | For the construction and optimization of acetyl-CoA biosynthesis |
| pYLXP'- tYlHMG1-YlPOT1-YlPAT1-YlACS2 | For the construction and optimization of aacetyl-CoA biosynthesis |
| pYLXP'- tYlHMG1-YlPOT1-YlPAT1-YlMFE2-YlACS2 | For the construction and optimization of acetyl-CoA biosynthesis |
| pYLXP'- EcispA-YlGGPP-YlERG20-YlERG8-YlERG12-YlMVD1-YlUPC2 | For the construction and optimization of mevalonate pathway |

(1) The subscripts “_x3_” refer to gene copy number in the plasmids.

**Supplementary figures**

Figure S1. Amorphadiene production through original and ER-tagged ADS gene.

# References

Basson, M. E., Thorsness, M., & Rine, J. (1986). Saccharomyces cerevisiae contains two functional genes encoding 3-hydroxy-3-methylglutaryl-coenzyme A reductase. *Proc Natl Acad Sci U S A*, *83*(15), 5563-5567.

Huang, Y. Y., Jian, X. X., Lv, Y. B., Nian, K. Q., Gao, Q., Chen, J., Wei, L. J., & Hua, Q. (2018). Enhanced squalene biosynthesis in Yarrowia lipolytica based on metabolically engineered acetyl-CoA metabolism. *J Biotechnol*, *281*, 106-114. <https://doi.org/10.1016/j.jbiotec.2018.07.001>

Marsafari, M., & Xu, P. (2020). Debottlenecking mevalonate pathway for antimalarial drug precursor amorphadiene biosynthesis in Yarrowia lipolytica. *Metabolic Engineering Communications*, *10*, e00121. <https://doi.org/https://doi.org/10.1016/j.mec.2019.e00121>

Martin, V. J., Pitera, D. J., Withers, S. T., Newman, J. D., & Keasling, J. D. (2003). Engineering a mevalonate pathway in Escherichia coli for production of terpenoids. *Nat Biotechnol*, *21*(7), 796-802. <https://doi.org/10.1038/nbt833>

Paddon, C. J., Westfall, P. J., Pitera, D. J., Benjamin, K., Fisher, K., McPhee, D., Leavell, M. D., Tai, A., Main, A., Eng, D., Polichuk, D. R., Teoh, K. H., Reed, D. W., Treynor, T., Lenihan, J., Fleck, M., Bajad, S., Dang, G., Dengrove, D., Diola, D., Dorin, G., Ellens, K. W., Fickes, S., Galazzo, J., Gaucher, S. P., Geistlinger, T., Henry, R., Hepp, M., Horning, T., Iqbal, T., Jiang, H., Kizer, L., Lieu, B., Melis, D., Moss, N., Regentin, R., Secrest, S., Tsuruta, H., Vazquez, R., Westblade, L. F., Xu, L., Yu, M., Zhang, Y., Zhao, L., Lievense, J., Covello, P. S., Keasling, J. D., Reiling, K. K., Renninger, N. S., & Newman, J. D. (2013). High-level semi-synthetic production of the potent antimalarial artemisinin. *Nature*, *496*(7446), 528-532. <https://doi.org/10.1038/nature12051>

Westfall, P. J., Pitera, D. J., Lenihan, J. R., Eng, D., Woolard, F. X., Regentin, R., Horning, T., Tsuruta, H., Melis, D. J., Owens, A., Fickes, S., Diola, D., Benjamin, K. R., Keasling, J. D., Leavell, M. D., McPhee, D. J., Renninger, N. S., Newman, J. D., & Paddon, C. J. (2012). Production of amorphadiene in yeast, and its conversion to dihydroartemisinic acid, precursor to the antimalarial agent artemisinin. *Proc Natl Acad Sci U S A*, *109*(3), E111-118. <https://doi.org/10.1073/pnas.1110740109>
